# Breastfeeding during the COVID-19 pandemic and associated factors: a risk to sustainable development

**DOI:** 10.1590/0034-7167-2024-0357

**Published:** 2025-12-15

**Authors:** Bárbara Portela Diniz, Danton Matheus de Souza, Sandra Josefina Ferraz Ellero Grisi, Ana Paula Scoleze Ferrer

**Affiliations:** I Universidade de São Paulo. São Paulo, São Paulo, Brazil

**Keywords:** Pandemics, COVID-19, Breast Feeding, Maternal and Child Health, Sustainable Development., Pandemias, COVID-19, Lactancia Materna, Salud Materno-Infantil, Desarrollo Sostenible.

## Abstract

**Objectives:**

to assess breastfeeding during the first six months of life of children born during the COVID-19 pandemic, identify associated factors and discuss them based on the Sustainable Development Goals.

**Method:**

a longitudinal study conducted with 127 mother-baby dyads born in the pre-vaccination phase of the COVID-19 pandemic, followed from birth to the sixth month of life. Variables for dyad identification, postpartum depression, bonding, feeding and breastfeeding practices were collected, with data subjected to descriptive and inferential analysis.

**Results:**

a high percentage of early weaning was observed, especially in the first month, associated with the use of a pacifier (p=0.019), difficulty breastfeeding (p=0.015), both in the first month of life, early weaning (p=0.001) and early formula introduction (p=0.001).

**Conclusion:**

shorter breastfeeding time and high percentage of early weaning observed in children born during the pandemic may compromise the achievement of the Sustainable Development Goals.

## RESUMEN

**Objetivos:**

evaluar la lactancia materna durante los primeros seis meses de vida de los niños nacidos durante la pandemia de COVID-19, identificar los factores asociados y discutirlos con base en los Objetivos de Desarrollo Sostenible.

**Método:**

estudio longitudinal, realizado con 127 díadas madre-bebé nacidas en la fase prevacunal de la pandemia de COVID-19, seguidas desde el nacimiento hasta el sexto mes de vida. Se recolectaron variables para identificar la díada, depresión puerperal, vínculo, prácticas alimentarias y de lactancia materna, siendo los datos sometidos a análisis descriptivo e inferencial.

**Resultados:**

se observó un alto porcentaje de destete precoz, especialmente en el primer mes, asociado al uso de chupete (p=0,019), dificultad para la lactancia materna (p=0,015), tanto en el primer mes de vida, destete precoz (p=0,001) e introducción precoz de fórmula (p=0,001).

**Conclusión:**

la menor duración de la lactancia materna y el alto porcentaje de destete precoz observado en los niños nacidos durante la pandemia pueden comprometer el logro de los Objetivos de Desarrollo Sostenible.

## INTRODUCTION

The Sustainable Development Goals (SDGs) aim to ensure healthy lives and promote well-being for all at all ages. In this context, special attention must be paid to maternal and child health, with a special focus on breastfeeding, a practice that is crucial to achieving the global agenda^(1)^. Breastfeeding is directly or indirectly related to the 17 SDGs. The International Network for the Education of Health Technicians, linked to the *Fundação Oswaldo Cruz*, published a note in 2016 listing the benefits of breastfeeding for achieving the SDGs^(2)^.

The most obvious link is between breastfeeding and SDG 2 (“End hunger, achieve food security and improved nutrition and promote sustainable agriculture”). Breast milk ensures adequate nutrition and is intrinsically linked to food security, particularly in environments of poverty and high rates of malnutrition^(3,4)^. SDG 3 includes nine targets aimed at “Ensuring healthy lives and promoting well-being for all at all ages”. In this regard, there is a wealth of scientific evidence demonstrating the relationship between breast milk and better health and well-being of individuals, such as: reduced infant mortality; lower incidence of infectious diseases; reduced prevalence of deficiency anemia and micronutrient deficiencies; protection against allergic diseases; reduced risk of obesity and metabolic syndrome; and promotion of better child development, bringing benefits to maternal health^(5-8)^.

In addition to the benefits mentioned above, breastfeeding is related to several aspects of socioeconomic and environmental sustainability, addressed by SDGs 5 (“Achieve gender equality and empower all women and girls”), 12 (“Ensure sustainable consumption and production patterns”) and 13 (“Take urgent action to combat climate change and its impacts”). Breastfeeding increases maternal autonomy, favoring their role in society and contributing to the promotion of gender equality, as long as the legal and labor guarantees related to the practice are respected, promoting social equality^(9,10)^. Indirectly, breastfeeding is associated with sustainable consumption patterns, production and climate change, since it does not require packaging, transportation or processing, and does not produce environmental waste^(11)^.

It is clear that breastfeeding is aligned with the SDGs. However, this practice is influenced by numerous circumstances, and healthcare professionals must be aware of these in order to promote it and act in situations that put its continuity at risk. Factors related to maternal health, child health, care practices and socio-environmental conditions can affect breastfeeding, and one example is the coronavirus disease 2019 (COVID-19) pandemic.

The pandemic has had a profound impact on global progress towards achieving the SDGs, as it has affected health, economic, social and environmental systems. Numerous undesirable effects have emerged, such as increased poverty, social inequality, food insecurity, and health conditions and well-being of the population^(12-14)^. In the absence of effective treatment and with high morbidity and mortality rates, the pre-vaccination period was marked by restrictive measures and social distancing.

Reduced access to healthcare services, including prenatal care and early first consultations, changes in care protocols during birth, such as the absence of skin-to-skin contact and separation between mothers and newborns, concerns related to the risk of transmission via breast milk, stress and reduced family support for postpartum women, have put the initiation and maintenance of breastfeeding at risk in the pandemic context^(15,16)^. These changes in care practices and the possible repercussions on maternal mental health have had an impact on breastfeeding. However, studies conducted in several countries have shown controversial results on this subject^(16)^.

A systematic review demonstrated that the pandemic led to variations in breastfeeding duration and daily frequency, while some articles reported an increase in breastfeeding time due to the greater presence of mothers at home. Others described that breastfeeding was negatively affected^(16)^. Despite the declaration of the end of the pandemic, its influences may permeate global health in the long term. Thus, a look at this context is emerging to understand its medium and long-term effects on breastfeeding and global development goals, advancing and contributing to the frontier of international knowledge.

### Study Relevance

This article is the product of the master’s dissertation^(17)^, titled “*Repercussões da pandemia no vínculo mãe-filho e no aleitamento materno durante o primeiro semestre de vida*”, deposited in the institutional repository: https://doi.org/10.11606/D.5.2023.tde-13112023-173529.

## OBJECTIVES

To assess breastfeeding during the first six months of life of children born during the COVID-19 pandemic, identify associated factors and discuss them based on the SDGs.

## METHODS

### Ethical aspects

This study respected the ethical guidelines set out in Resolutions 466/12 and 510/16 of the Brazilian National Health Council, as well as followed the guidelines for conducting research in a virtual environment of Circular Letter 02/2021 of the Brazilian National Research Ethics Committee^(18)^. Furthermore, it received approval from the Research Ethics Committee.

### Study design

This is an observational, longitudinal and quantitative study. The STrengthening the Reporting of OBservational studies in Epidemiology recommendations were followed for its writing^(19)^.

### Study location, period and sample

The study was conducted at a teaching hospital that assists a population dependent on the Brazilian Health System in the state of São Paulo, where only children born to uninfected pregnant women were born. A convenience sample was composed of postpartum women who were on the first day postpartum in rooming-in unit on the days the researcher visited: Mondays, Wednesdays, and Fridays, from February 7, 2021 to June 1, 2021, including pregnancies that occurred entirely during the first 18 months of the pandemic. This period was characterized by greater social restrictions, and immunization was not universally available. It is noteworthy that, in São Paulo, vaccination began on January 17, 2021 for healthcare professionals, but it only became available for pregnant and postpartum women with comorbidities on May 11, and for those without comorbidities, on June 7, 2021; therefore, after the end of data collection.

### Inclusion and exclusion criteria

Mother-baby dyads who were rooming-in and agreed to participate after signing the Informed Consent Form were included. Those who had any condition that contraindicated or could interfere with breastfeeding were excluded: twin births; malformed children; children who had remained in the Neonatal Intensive Care Unit in the first 24 hours of life; children of mothers with pathologies or using medications who had been advised not to breastfeed; and those who did not respond to the researchers in the virtual follow-up contacts, conducted on three different days and times.

### Study protocol

Data were collected by a researcher, nutritionist and master’s student in pediatrics, with the assistance of a previously trained nursing student in scientific initiation, with an in-person interview being conducted on the first day after birth in rooming-in unit. Subsequent follow-up was carried out via telephone contact and through a messaging application, initially between the 15^th^ and 20^th^ day of life, followed by monthly contacts, until the sixth and seventh month of life.

A semi-structured questionnaire developed by the researchers was used to characterize maternal characteristics, birth conditions and information about prenatal care, pregnancy and puerperium. The Edinburgh Postnatal Depression Scale (EPDS) was used to screen for postpartum depression, and the Postpartum Bonding Questionnaire (PBQ) was used to characterize the mother-child bond.

The EPDS was administered in the first month of life, chosen because it is easy to apply and interpret and because it has been adapted and validated in Brazil. A score greater than or equal to 10 was adopted to characterize probable postpartum depression, as it is the most widely used cut-off point in national literature, allowing comparison among studies^(20,21)^. The PBQ was sent via messaging app in the first and last months of follow-up. This scale allows assessing the mother-baby bond in the postpartum period, and is considered a reliable instrument for detecting dysfunctions in the mother-child relationship, and has been translated, adapted and validated in Brazil. It consists of 25 questions and assesses four mother-child relationship domains: general bonding disorders (12 items); severe mother-infant relationship disorders (seven items); infant-focused anxiety (four items); and risk of abuse (two items). For this study, we considered only the responses from the general bonding disorders domain, which presents the best psychometric indices^(22-24)^.

Furthermore, in monthly telephone contacts, the researchers asked about breastfeeding maintenance, food introduction, and, in the case of weaning, the reason was asked.

### Analysis of results, and statistics

The data were subjected to descriptive and inferential analysis, with associations between independent variables and breastfeeding duration, using nonparametric techniques, since the data normality was rejected by the Shapiro-Wilk test. Thus, the Kendall Tau-b correlation test was used for continuous variables, and Fisher’s exact test was used for categorical variables. For the correlation between breastfeeding duration and bonding scores in the first and sixth months of life, the Kendall Tau-b correlation test was used. The p-value<0.05 was considered as statistical significance. Furthermore, for the discussion, the findings are articulated with sustainable development^(1)^.

## RESULTS

During the data collection period, 621 births were performed, of which 288 dyads were eligible, who were on the first day postpartum. Of these, 32 refused to participate and 78 were excluded (49 due to maternal causes and 29 related to newborns’ condition). A total of 178 interviews were conducted in the immediate postpartum period. During the longitudinal follow-up, in the first six months of life, there were 51 losses to follow-up, totaling 127 mother-infant dyads in the final sample.

The mean maternal age was 27 years, ranging from 18 to 43 years. The majority had completed high school (70.9%), were married or lived with a partner (72.4%) and were multiparous (59.8%). Although 70.9% of women had not planned their pregnancy, only one reported not having attended prenatal care. In the EPDS, answered one month after delivery, 29.2% of postpartum women had a risk score for probable postpartum depression. Regarding babies, there was a slight predominance of females (55.1%), with half being born by vaginal delivery (50.4%) and with adequate weight (average of 3.3 kg), and only four (3.1%) were premature (Table 1).

**Table 1:** Sample characterization (N=127). São Paulo, SP, Brazil, 2021

MATERNAL CHARACTERISTICS		
Age (years)	**Min.-Max.**	**Mean (SD) - Median (95%CI)**
	18 - 43	27.6 (6.0) - 28 (26.5 - 28.6)
Education	**N**	**%**
Up to elementary school	13	10.2
High school	90	70.9
Higher education	24	18.9
Marital status	**N**	**%**
Without a partner	35	27.6
With a partner	92	72.4
Planned pregnancy	**N**	**%**
Yes	37	29.1
No	90	70.9
Had prenatal care	**N**	**%**
Yes	126	99.2
No	1	0.8
Parity	**N**	**%**
Primiparous	51	40.2
Multiparous	76	59.8
EPDS score	**Min.-Max.**	**Mean (SD) - Median (95%CI)**
	0 - 26	7.8 (5.3) - 7.0 (6.9 - 8.8)
EPDS classification	**N**	**%**
Normal	89	70.0
Probable postpartum depression	37	29.2
No information	1	0.8
**NEWBORN CHARACTERISTICS AND BIRTH CONDITIONS**		
Sex	**N**	**%**
Male	57	44.9
Female	70	55.1
Type of childbirth	**N**	**%**
Vaginal	64	50.4
Cesarean	63	49.6
Gestational age	**N**	**%**
Term	121	95.3
Preterm	4	3.1
No information	2	1.6
Birth weight (kg)	**Min.-Max.**	**Mean (SD)**
	2.3 - 4.3	3.3 (4.0)
Pacifier use in the first month of life	**N**	**%**
Yes	51	40.2
No	76	59.8

*Note: SD - standard deviation; 95%CI - 95% Confidence Interval; Min-Max - Minimum-Max; EPDS - Edinburgh Postnatal Depression Scale.*

**Figure 1:**
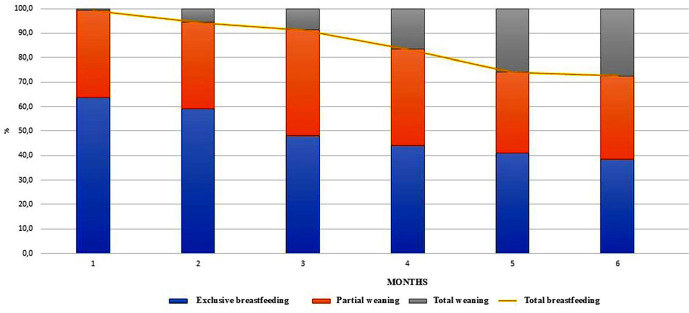
Evolution of breastfeeding time (N=127). São Paulo, SP, Brazil, 2023

Most mothers received guidance on breastfeeding in rooming-in (83.5%) and not during prenatal care (21.2%), and only 36.2% of newborns were breastfed in the delivery room. Almost half of newborns had difficulty breastfeeding in rooming-in (48%), with this percentage improving during the first month of life (17.3%), and only seven needed to receive formula in the maternity ward. Only 63.8% of newborns were exclusively breastfed at the end of the first month of life, and formula was introduced before 6 months of age in 48.8% of babies. The total breastfeeding time was 5.2 months, and 3.2 months when exclusive breastfeeding was considered (Table 2). Figure 1 shows the evolution of breastfeeding time in the sample studied.

**Table 2:** Feeding practices and breastfeeding duration. São Paulo, SP, Brazil, 2021

Did the mother receive guidance on breastfeeding during prenatal care?	N	%
Yes	27	21.2
No	100	78.8
Did the mother receive guidance on breastfeeding in rooming-in?	**N**	**%**
Yes	106	83.5
No	21	16.5
Did the newborn breastfeed in the delivery room?	**N**	**%**
Yes	46	36.2
No	81	63.8
Did the newborn have difficulty breastfeeding in rooming-in?	**N**	**%**
Yes	61	48.0
No	66	52.0
Did the newborn receive formula in rooming-in?	**N**	**%**
Yes	7	5.5
No	120	94.5
Did the newborn have difficulty breastfeeding in the first month of life?	**N**	**%**
Yes	22	17.3
No	105	82.7
Breastfeeding in the first month of life	**N**	**%**
Exclusive	81	63.8
Partial weaning	45	35.4
Total weaning	1	0.8
Age of formula introduction	**N**	**%**
< 3 months	17	13.4
≥ 3-6 months	45	35.4
≥ 6 months/not introduced	65	51.2
Total breastfeeding time (months)	**Min.-Max.**	**Mean (SD) - Median (95%CI)**
	0->6	5.2 (1.5) - 6.0 (4.9 - 5.4)
	**N**	**%**
< 3 months	11	8.7
≥ 3-6 months	24	18.9
≥ 6 months	92	72.4
Exclusive breastfeeding time (months)	**Min.-Max.**	**Mean (SD) - Median (95%CI)**
	0 - 6	3.2 (2.3) - 4.0 (2.8 - 3.6)
	**N**	**%**
< 3 months	50	39.4
≥ 3-6 months	28	22.0
≥ 6 months	49	38.6

*Note: SD - standard deviation; 95%CI - 95% confidence interval; Min-Max - Minimum-Maximum.*

**Figure 2:**
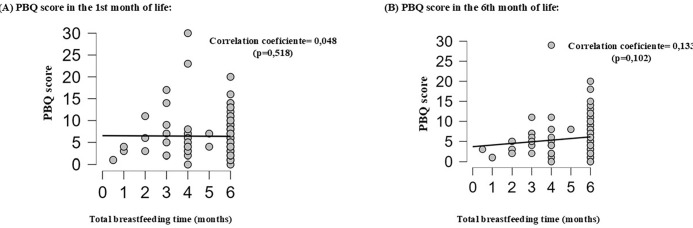
Correlation between total breastfeeding duration and mother-infant bonding scores in the first (A) and sixth month of life (B) (N=127). São Paulo, SP, Brazil, 2021

From inferential analysis, factors associated with breastfeeding time were observed, such as pacifier use in the first month of life (p=0.019), difficulty breastfeeding in the first month of life (p=0.015), early weaning and early formula introduction (p<0.001) (Table 3). Figure 2 represents the correlation between total breastfeeding time and bonding scores obtained by the PBQ in the first (p=0.518) and sixth month of life (p=0.102).

**Table 3:** Factors associated with total breastfeeding duration (N=127). São Paulo, SP, Brazil, 2021

	< 3 months	≥ 3-6 months	≥ 6 months	Coefficient	*p* value
Maternal age	-	-	-	0.097	0.182
Education					
Up to elementary school	1(7.7)	3(23.1)	9(69.2)	0.058	0.491
High school	8(8.9)	18(20.0)	64(71.1)		
Higher education	2(8.3)	3(12.5)	19(79.2)		
Marital status					
Without a partner	3(8.6)	9(25.7)	23(65.7)	-0.081	0.348
With a partner	8(8.7)	15(16.3)	69(75.0)		
Planned pregnancy					
Yes	2(5.4)	4(10.8)	31(83.8)	0.155	0.073
No	9(10.0)	20(22.2)	61(67.8)		
Had prenatal care					
Yes	11(8.7)	23(18.3)	92(73.0)	-	-
No	0	1(100.0)	0		
Parity					
Primiparous	4(7.8)	8(15.7)	39(76.5)	0.069	0.427
Multiparous	7(9.2)	16(21.1)	53(69.7)		
No	1(5.9)	4(23.5)	12(70.6)		
EPDS classification					
Normal	9(10.1)	19(21.4)	61(68.5)	0.125	0.152
Probable postpartum depression	2(5.4)	5(13.5)	30(81.1)		
Sex					
Male	5(8.8)	11(19.3)	41(71.9)	-0.010	0.911
Female	6(8.6)	13(18.6)	51(72.8)		
Type of delivery					
Vaginal	4(6.2)	16(25.0)	44(68.8)	-0.059	0.498
Caesarean section	7(11.1)	8(12.7)	48(76.2)		
Gestational age					
Preterm	0	1(25.0)	3(75.0)	-	-
Term	11(9.1)	22(18.2)	88(72.7)		
Pacifier use in the first month of life					
Yes	6(11.8)	14(27.4)	31(60.8)	-0.203	0.019
No	5(6.6)	10(13.1)	61(80.3)		
Guidance on breastfeeding during prenatal care					
Yes	2(7.4)	7(25.9)	18(66.7)	-0.055	0.525
No	9(9.0)	17(17.0)	74(74.0)		
Guidance on breastfeeding in rooming-in					
Yes	8(7.6)	21(19.8)	77(72.6)	0.024	0.784
No	3(14.3)	3(14.3)	15(71.4)		
Breastfeeding in the delivery room					
Yes	3(6.5)	11(23.9)	32(69.6)	-0.033	0.703
No	8(9.9)	13(16.0)	60(74.1)		
Difficulty breastfeeding in rooming-in					
Yes	8(13.1)	12(19.7)	41(67.2)	-0.124	0.152
No	3(4.5)	12(18.2)	51(77.3)		
Difficulty breastfeeding in the first month of life					
Yes	6(27.3)	4(18.2)	12(54.5)	-0.211	0.015
No	5(4.8)	20(19.0)	80(76.2)		
Exclusive breastfeeding in the first month of life					
Yes	2(2.5)	12(14.8)	67(82.7)	-0.317	<0.001
No	9(19.6)	12(26.1)	25(54.3)		
Age of formula introduction					
< 3 months	10(58.8)	4(23.6)	3(17.7)	-0.640	<0.001
≥ 3-6 months	0	20(44.4)	25(55.6)		
≥ 6 months/not introduced	1(1.5)	0	64(98.5)		

*Note: EPDS - Edinburgh Postnatal Depression Scale.*

## DISCUSSION

In this study, 72.4% of mothers continued breastfeeding until their children were six months old, but only 38.6% were exclusively breastfeeding, as recommended. This rate is below the 45.8% described in the Brazilian National Study on Infant Feeding and Nutrition (In Portuguese, *Estudo Nacional de Alimentação e Nutrição Infantil* - ENANI)^(25)^, the 2020 global indicator (44%) and well below the target recommended by the World Health Organization (WHO) of 50% in 2025 and 70% in 2030^(26)^, showing failures in breastfeeding promotion, considered one of the pillars for achieving the SDGs, particularly SDGs 2 and 3, related to individuals’ nutritional and health status, but indirectly also to the SDGs related to socioeconomic and environmental issues (SDGs 5, 12 and 13).

Another relevant result was the high percentage of children (36.2%) who were not exclusively breastfed in the first month of life, mainly because this result reflected the total breastfeeding time. While 82.7% of children who were exclusively breastfed in the first month of life had a total breastfeeding time greater than six months, only 54.3% of those who were not breastfed received breast milk for more than six months (p<0.001). It is important to highlight that 46 children were not exclusively breastfed in the first month of life, but formula consumption before three months was reported by a much smaller number (17 mothers). This data shows us that weaning may not have been due to the early use of formula, but rather to the introduction of other liquids, such as water, juice and tea, and that this ended up interfering with the total breastfeeding time. This is a common practice and should be discouraged. A similar result was described by other authors, and one of the explanations would be interference with the sucking habit^(27)^. Problems with breastfeeding technique were possibly one of the main reasons for early weaning, as reported difficulty breastfeeding in the first month of life was also associated with shorter total breastfeeding time (p=0.015). Exclusive breastfeeding up to six months of age has been associated with reduced maternal and infant morbidity and mortality^(5-8)^. Therefore, the early weaning identified in this study may pose a threat to the achievement of SDGs 2 and 3.

In addition to being associated with early weaning, the provision of water, juices and teas does not meet newborns’ and young infants’ nutritional needs, but increases the risk of contamination, particularly in conditions of less hygiene^(28)^, compromising food security and nutritional status (SDG 2)^(3)^. These results demonstrate the importance of early childcare consultations in the first few days after discharge from the maternity ward so that breastfeeding can be assessed and mothers can be adequately guided on the technique and practice of introducing liquids other than breast milk.

Proper guidance and support for women is essential for successful breastfeeding. Female empowerment is correlated with breastfeeding as a “two-way street”: women who feel safe have a greater chance of successful breastfeeding; on the other hand, successful breastfeeding encourages maternal autonomy^(9,10)^, promoting the achievement of SDG 5. In this sense, another result that draws attention in this study is that, although practically all pregnant women had prenatal care, the majority (78.8%) reported that they did not receive guidance on breastfeeding during this follow-up. It is known that the decision to breastfeed a child or not occurs, in most cases, well before birth, and the guidance provided during prenatal care contributes to women’s decision to breastfeed and its duration^(29)^. On the other hand, 83.5% of the mothers studied reported having received guidance from rooming-in nurse, and this may have influenced the decrease in the percentage of newborns with difficulty breastfeeding: from 48% in the maternity ward to 17.3% after hospital discharge. This guidance may have contributed to the total breastfeeding time, although no statistical significance was found.

The aforementioned reflections demonstrate the need for empowerment of healthcare services, especially primary care, which plays an active role in prenatal care, home visits and childcare consultations, and healthcare professional empowerment, making use of these devices for their work in breastfeeding, consequently impacting the SDGs.

Another finding that reinforces the importance of guidance and the relationship between practices that interfere with sucking and breastfeeding habits was the association between early weaning and use of pacifiers in the first month of life (p=0.019). In this study, 40.2% used pacifiers, similar to the 43.9% described in ENANI^(25)^. Pacifiers are artificial nipples made of rubber or silicone that are designed to provide comfort and satisfy the sucking reflex in infants. Their use has been associated with a number of potential benefits, including reduced risk of sudden infant death syndrome and pain relief during invasive manipulations. However, pacifiers have been associated with negative outcomes, including decreased breastfeeding rates and duration^(30)^, as seen in the results. Research suggests that pacifier use may interfere with an infant’s ability to suck effectively, leading to nipple confusion, decreased milk production, and shorter breastfeeding duration^(31)^, but these findings remain inconsistent. Other authors have not found this impact of pacifier use on breastfeeding outcomes^(32)^. In any case, the American Academy of Pediatrics recommends delaying pacifier use until breastfeeding is well established, usually around 3-4 weeks of age, as does the Baby-Friendly Hospital Initiative, a global program to encourage breastfeeding that also discourages routine pacifier use in infants^(33)^.

Regarding early formula introduction, we also found an association with the total breastfeeding time (p<0.001). Thus, it is worth considering that it is difficult to identify what was the cause and what was the consequence, i.e., whether the children who received formula breastfed for less time or vice versa. In any case, this is a finding also discussed in literature^(34)^, particularly when this introduction is very early, in the first days of life.

Previous research has found that formula introduction in hospital was associated with shorter duration of exclusive breastfeeding. Reported reasons include interference with sucking ability, reduced milk production due to reduced frequency of feedings, and possible changes in maternal perception of the sufficiency of breast milk, as they tend to view their milk supply as inadequate and stop breastfeeding early^(34)^. Hence, it was not possible to analyze the impact of introduction in the first three days of life, as described by some authors, because there was a small number of children who received formula in the maternity ward (5.5%). However, it was found that the earlier the formula introduction, the shorter the total breastfeeding time.

Although no association was observed between early weaning and the presence of probable postpartum depression, as frequently reported in literature, the high percentage (29.2%) of postpartum women with a risk score is noteworthy. Research carried out before the pandemic, involving 2,687 women and using the same screening instrument and cut-off score, described a prevalence of 14%^(35)^. The results indicated that the risk of postpartum depression doubled during the pandemic. Other authors^(36)^ found even higher rates in Brazil (38.8%) and Spain (37.3%)^(37)^. These findings highlight the need for healthcare professionals who care for postpartum women and infants to take a more cautious and attentive look at this issue. Routinely incorporating screening scales for maternal depression into care protocols should be considered by healthcare services. This worsening of mental health was widespread during the pandemic, and is one of the points considered most impactful for achieving SDG 3^(12,13)^.

Postpartum depression is associated with several negative outcomes, such as worsening of the mother-baby bond. In our study, 9.4% of the sample presented altered scores on the PBQ performed in the first month of life, indicating that the bond between the dyad was altered early on. This finding is possibly due to the negative effects of the pandemic on mental health. A study that compared two samples of mothers before and during the pandemic found that they were 2.56 times more likely to have an altered score in the bond assessment one month after birth^(38)^. This study supports another investigation, which found a worsening in PBQ scores carried out in the first year of life in Portuguese mothers, relating worst scores to parental stress^(39)^. The researchers did not have data available prior to the pandemic, and it is not possible to state whether there was a cause-and-effect relationship. However, the high prevalence of postpartum depression may explain this unfavorable result. Another aspect that could explain these altered PBQ scores in the first month of life is the fact that only 36.2% of newborns were breastfed in the delivery room. It is known that this skin-to-skin contact is widespread and encouraged to favor the bond between mother and child^(40)^, but we must consider that this study took place in the phase of greatest restriction, during the pandemic, in which care protocols in the delivery room had to be modified.

Although there was no statistical significance, a correlation was observed between the PBQ score, both in the first and sixth months of life, and breastfeeding duration, indicating that greater bonding was associated with longer breastfeeding duration. The lack of statistical significance indicates that the relationship between the two is complex and that multiple factors are involved in establishing the mother-child bond and in the success of breastfeeding.

In 2018, the WHO released the Nurturing Care initiative, which should guide child healthcare. Its essential components include health promotion, adequate nutrition, responsive care, safety and providing opportunities for child development. These components are interconnected with the 17 SDGs and are central to the reflection of strategies for sustainable development^(41)^. Breastfeeding is integrated into all of the above components, as indicated throughout this manuscript. Threats to its maintenance can impact the global health of children and, consequently, humanity, reinforcing the importance of looking at the phenomenon and guiding new research that aims to change this scenario.

It should be noted that, in this study, although most children were breastfed for six months or more, the rate of early weaning, from the first month onwards, was very high. This result may represent barriers to achieving the SDG targets, especially zero hunger (SDG 2), good health and well-being (SDG 3), gender equity (SDG 5), responsible consumption and production (SDG 12), and climate action (SDG 13).

### Study limitations

Since this was a six-month remote follow-up study conducted during the pandemic, sample loss may be a result of selection bias and a limitation, since the losses were possibly among mothers with worse outcomes because they did not respond to the questionnaires. Another limitation is the lack of possibility of establishing causal relationships between the findings and the pandemic and direct relationships with the SDGs. Despite this, it was possible to characterize the practice of breastfeeding, probable postpartum depression, and the bond during the most critical period of this historical moment, with discussions regarding the SDGs.

### Contributions to nursing, health or public policy

This study demonstrates the importance of maternal and child care and the need for a cautious and attentive approach by nurses and other healthcare professionals who work with pregnant women, postpartum women, and infants. The factors associated with early weaning are possibly a reflection of the lack of guidance and support that postpartum women received during the pandemic, due to social distancing and reduced access to childcare consultations. These findings demonstrate that, in order to promote exclusive breastfeeding, an important pillar for achieving the SDGs, feasible measures are needed, through quality maternal and child care. Furthermore, the innovative nature of this manuscript stands out, as it addresses a phenomenon of international importance in a critical period for public health and its articulations with the SDGs, demonstrating that, even with the end of the pandemic period, efforts must be made to mitigate the possible long-term impacts caused by this context.

## CONCLUSIONS

In the first six months of life, the total duration of breastfeeding was 5.2 months, and exclusive breastfeeding was 3.2 months. Although 72.4% continued breastfeeding until the sixth month of life, only 36.2% were exclusively breastfed in the first month of life, characterizing a high percentage of early weaning during the COVID-19 pandemic. These results point to greater nutritional and health risks among these children born during the pandemic and, therefore, with a possible impact on the achievement of SDGs 2 and 3. Likewise, the early weaning observed may indirectly harm the achievement of SDGs 5, 12 and 13 by reducing the socioeconomic and environmental benefits associated with breastfeeding.

By assessing the factors that were associated with shorter total breastfeeding time, this study provides insights to improve healthcare practices. The factors identified were pacifier use (p=0.019), difficulty breastfeeding (p=0.015), both in the first month of life, early weaning (p=0.001) and early formula introduction (p=0.001), reinforcing the importance of improving healthcare aimed at promoting breastfeeding, in order to mitigate the undesirable effects for achieving the SDGs in the post-pandemic period.

## Data Availability

Research data is only available upon request.
