# Supplementary material for: Breastfeeding during the COVID-19 pandemic and associated factors: a risk to sustainable development
Source: Rev Bras Enferm. 2025 Dec 15;78(5):e20240357. doi: 10.1590/0034-7167-2024-0357 (PMC12707855; doi:10.1590/0034-7167-2024-0357)
Supplement: Translation

# Translation: Aleitamento materno durante a pandemia de COVID-19 e fatores associados: um risco ao desenvolvimento sustentável

**DOI:** 10.1590/0034-7167-2024-0357pt

**Authors:** Bárbara Portela Diniz, Danton Matheus de Souza, Sandra Josefina Ferraz Ellero Grisi, Ana Paula Scoleze Ferrer

**Affiliations:** I Universidade de São Paulo. São Paulo, São Paulo, Brasil

**Keywords:** Pandemias, COVID-19, Aleitamento Materno, Saúde Materno-infantil, Desenvolvimento Sustentável.

## Abstract

**Objetivos:**

avaliar o aleitamento materno durante o primeiro semestre de vida de crianças nascidas durante a pandemia de COVID-19, identificar os fatores associados e discuti-los com base nos Objetivos De Desenvolvimento Sustentável.

**Método:**

estudo longitudinal, conduzido com 127 díades mães-bebês nascidos na fase pré-vacinação da pandemia de COVID-19, acompanhadas do nascimento ao sexto mês de vida. Coletaram-se variáveis de identificação da díade, depressão puerperal, vínculo, práticas alimentares e de aleitamento materno, com dados submetidos à análise descritiva e inferencial.

**Resultados:**

observou-se um elevado porcentual de desmame precoce, especialmente no primeiro mês, associado ao uso de chupeta (p=0,019), dificuldade para mamar (p=0,015), ambos no primeiro mês de vida, desmame precoce (p=0,001) e introdução precoce de fórmula (p=0,001).

**Conclusão:**

o menor tempo de aleitamento materno e o elevado porcentual de desmame precoce observados nas crianças nascidas durante a pandemia podem comprometer o alcance dos Objetivos De Desenvolvimento Sustentável.

## INTRODUÇÃO

Os Objetivos de Desenvolvimento Sustentável (ODS) visam assegurar vidas saudáveis e promover o bem-estar para todos, em todas as idades. Nesse contexto, atenção especial deve se voltar à saúde materno-infantil, com um enfoque especial para o aleitamento materno, prática crucial para o alcance da agenda global^(1)^. O aleitamento materno se relaciona com os 17 ODS, de forma direta ou indireta. A Rede Internacional de Educação de Técnicos em Saúde, ligada à Fundação Oswaldo Cruz, publicou em 2016 uma nota em que relaciona os benefícios do aleitamento materno para o alcance dos ODS^(2)^.

A relação mais evidente é percebida entre o aleitamento materno e o ODS 2 (“Acabar com a fome, alcançar a segurança alimentar e melhoria da nutrição e promover a agricultura sustentável”). O leite materno garante a nutrição adequada e está intrinsecamente relacionado à segurança alimentar, particularmente em ambientes de pobreza e altos índices de desnutrição^(3,4)^. O ODS 3 inclui nove metas visando “Assegurar uma vida saudável e promover o bem-estar para todas e todos, em todas as idades”. Nesse sentido, há inúmeras evidências científicas demonstrando a relação entre o leite materno e melhores condições de saúde e bem-estar dos indivíduos como: a redução da mortalidade infantil; a menor incidência de doenças infecciosas; a redução da prevalência de anemia carencial e déficits de micronutrientes; a proteção contra doenças alérgicas; a diminuição do risco de obesidade e síndrome metabólica; e a promoção do melhor desenvolvimento infantil, trazendo benefícios para a saúde materna^(5-8)^.

Além dos benefícios supracitados, o aleitamento materno se relaciona a diversos aspectos de sustentabilidade socioeconômica e ambiental, abordados pelos ODS 5 (“Alcançar a igualdade de gênero e empoderar todas as mulheres e meninas”), 12 (“Assegurar padrões de produção e de consumo sustentáveis”) e 13 (“Tomar medidas urgentes para combater a mudança climática e seus impactos”). A amamentação aumenta a autonomia materna, favorecendo o seu papel na sociedade e contribuindo para a promoção da equidade de gênero, desde que respeitadas as garantias legais e trabalhistas relacionadas à prática, promovendo a igualdade social^(9,10)^. De forma indireta, o aleitamento materno se associa a padrões de consumo, produção sustentáveis e mudanças climáticas, uma vez que que não requer embalagens, transporte ou processamento, e não produz resíduos ambientais^(11)^.

É evidente que o aleitamento materno está alinhado às metas do desenvolvimento sustentável. Contudo, essa prática sofre a influência de inúmeras circunstâncias, e os profissionais de saúde devem conhecê-las para promovê-lo e atuar em situações que coloquem a sua continuidade em risco. Fatores relacionados à saúde materna, à criança, às práticas assistenciais e às condições socioambientais podem afetar o aleitamento, e um exemplo é a pandemia da doença do coronavírus 2019 (COVID-19).

A pandemia teve um impacto profundo no progresso mundial em direção ao alcance dos ODS, uma vez que afetou os sistemas de saúde, econômicos, sociais e ambientais. Inúmeros efeitos indesejáveis surgiram, como o aumento da pobreza, da desigualdade social, da insegurança alimentar, das condições de saúde e do bem-estar da população^(12-14)^. Na ausência de tratamento efetivo e com elevadas taxas de morbimortalidade, o período pré-vacinação foi marcado por medidas restritivas e de distanciamento social.

O menor acesso aos serviços de saúde, incluindo os atendimentos de pré-natal e de primeira consulta precoce, a modificação dos protocolos assistenciais durante o nascimento, como a ausência do contato pele a pele e a separação entre mães e recém-nascidos, as preocupações relacionadas ao risco de transmissão via leite materno, o estresse, e o menor suporte familiar às puérperas colocaram o início e a manutenção do aleitamento materno em risco no contexto pandêmico^(15,16)^. Essas mudanças nas práticas assistenciais e as possíveis repercussões na saúde mental materna tiveram impactos sobre o aleitamento materno. Entretanto, os estudos realizados em diversos países demonstraram resultados controversos em relação a esse assunto^(16)^.

Revisão sistemática demonstrou que a pandemia levou à variação do tempo de duração e da frequência diária de amamentação das crianças, enquanto alguns artigos relatavam aumento no tempo de aleitamento materno, devido à maior presença das mães em casa. E outros descreveram que a amamentação foi afetada negativamente^(16)^. Apesar da declaração do fim da pandemia, suas influências podem permear a longo prazo na saúde global. Assim, um olhar a esse contexto é emergente para compreender seus efeitos em médio e longo prazo na amamentação e nas metas mundiais de desenvolvimento, avançando e contribuindo à fronteira de conhecimento internacional.

### Relevância do estudo

Este artigo é produto da dissertação^(17)^, intitulada: “Repercussões da pandemia no vínculo mãe-filho e no aleitamento materno durante o primeiro semestre de vida”, depositada no repositório institucional: https://doi.org/10.11606/D.5.2023.tde-13112023-173529.

## OBJETIVOS

Avaliar o aleitamento materno durante o primeiro semestre de vida de crianças nascidas durante a pandemia de COVID-19, identificar os fatores associados e discuti-los com base nos ODS.

## MÉTODOS

### Aspectos éticos

Este estudo respeitou as diretrizes éticas pautadas nas Resoluções n° 466/12 e n° 510/16 do Conselho Nacional de Saúde, bem como seguiu as orientações para condução de pesquisas em ambiente virtual do Ofício Circular n° 02/2021 da Comissão Nacional de Ética em Pesquisa^(18)^. Ainda, recebeu aprovação pelo Comitê de Ética em Pesquisa.

### Delineamento do estudo

Trata-se de estudo observacional, longitudinal, de abordagem quantitativa. Para a sua redação, seguiram-se as recomendações do *STrengthening the Reporting of OBservational studies in Epidemiology* ^(19)^.

### Local, período e amostra do estudo

O estudo foi conduzido em hospital-escola que atende população dependente do Sistema Único de Saúde brasileiro no estado de São Paulo, no qual nasciam apenas crianças de gestantes não infectadas. Foi composta uma amostra de conveniência incluindo as puérperas que estavam no primeiro dia pós-parto no alojamento conjunto nos dias de visita da pesquisadora: às segundas, quartas e sextas-feiras, no período de 07 de fevereiro de 2021 a 01 de junho de 2021, incluindo gestações que ocorreram integralmente durante os primeiros 18 meses da pandemia. Esse período foi caracterizado por maiores restrições sociais, e a imunização não era disponível de maneira universal. É digno de nota que, em São Paulo, a vacinação começou em 17 de janeiro de 2021 para os profissionais de saúde, mas somente em 11 de maio ficou disponível para as gestantes e puérperas com comorbidade, e para as sem comorbidade, em 07 de junho de 2021; portanto, após a finalização da coleta de dados.

### Critérios de inclusão e exclusão

Foram incluídas as díades mães-bebês que estavam no alojamento conjunto e que aceitaram participar, após a assinatura do Termo de Consentimento Livre e Esclarecido. Foram excluídas as que possuíam alguma condição que contraindicasse ou que pudesse interferir com o aleitamento materno: parto gemelar; crianças malformadas; crianças que tenham permanecido em Unidade de Terapia Intensiva Neonatal nas primeiras 24 horas de vida; filhos de mães com patologias ou em uso de medicações que tenham sido orientadas a não amamentar; e as que não responderam aos pesquisadores nos contatos virtuais de seguimento, conduzidos em três dias e horários diferentes.

### Protocolo do estudo

Os dados foram coletados por uma pesquisadora, nutricionista e mestranda em pediatria, com auxílio de um discente de enfermagem, em iniciação científica, previamente treinado, sendo realizada uma entrevista presencial no primeiro dia pós-parto no alojamento conjunto. O acompanhamento subsequente foi realizado via contato telefônico e por meio de aplicativo de mensagens, realizados inicialmente entre o 15º e 20º dia de vida, seguidos por contatos mensais, até o sexto e sétimo mês de vida.

Para a caracterização sobre as características maternas, condições de nascimento e informações sobre o pré-natal, gestação e puerpério, foi aplicado um questionário semiestruturado elaborado pelos pesquisadores. Para o rastreio de depressão puerperal, foi utilizada a *Edinburgh Postnatal Depression Scale* (EPDS), e para caracterização do vínculo mãe-filho, utilizou-se o *Postpartum Bonding Questionnaire* (PBQ).

A EPDS foi aplicada no primeiro mês de vida, escolhida por ser de fácil aplicação e interpretação e por estar adaptada e validada no Brasil. Foi adotado o escore maior ou igual a 10 para caracterizar provável depressão puerperal, por ser o ponto de corte mais utilizado na literatura nacional, permitindo a comparação entre estudos^(20,21)^. Já o PBQ foi enviado via aplicativo de mensagens no primeiro e no último mês de acompanhamento. Essa escala permite avaliar o vínculo mãe-bebê no período pós-parto, sendo considerado um instrumento confiável para detectar disfunções na relação mãe-filho, e está traduzido, adaptado e validado no Brasil. É composta por 25 perguntas e avalia quatro domínios da relação mãe-filho: vínculo (12 itens); rejeição (sete itens); ansiedade relacionada à criança (quatro itens); e risco de abuso (dois itens). Para este estudo, consideramos apenas as respostas do domínio vínculo, o qual apresenta os melhores índices psicométricos^(22-24)^.

Ainda, nos contatos telefônicos mensais, os pesquisadores perguntaram sobre a manutenção do aleitamento materno, a introdução de alimentos, e, em caso de desmame, questionava-se o motivo.

### Análise dos resultados e estatística

Os dados foram submetidos à análise descritiva e inferencial, com associações entre as variáveis independentes e o tempo de aleitamento materno, utilizando-se técnicas não paramétricas, uma vez que a normalidade dos dados foi rejeitada pelo teste de Shapiro-Wilk. Dessa forma, utilizaram-se o teste de correlação de Tau-b de Kendall, para as variáveis contínuas, e o teste exato de Fisher, para as variáveis categóricas. Para a correlação entre o tempo de aleitamento materno e os escores de vínculo no primeiro e sexto mês de vida, utilizou-se o teste de correlação de Tau-b de Kendall. Considerou-se o valor de p<0,05 como significância estatística. Ainda, para a discussão, os achados são articulados com o desenvolvimento sustentável^(1)^.

## RESULTADOS

Durante o período da coleta de dados, foram realizados 621 partos, sendo elegíveis 288 díades, que estavam no primeiro dia pós-parto. Dessas, 32 recusaram a participação e 78 foram excluídas (49 devido a causas maternas e 29 relacionadas à condição do recém-nascido). Foram conduzidas 178 entrevistas no pós-parto imediato. Durante o seguimento longitudinal, no primeiro semestre de vida, houve 51 perdas de seguimento, totalizando 127 díades mães-bebês na amostra final.

A idade materna média foi de 27 anos, variando entre 18 e 43 anos. A maioria tinha ensino médio completo (70,9%), era casada ou morava junto com o companheiro (72,4%) e multípara (59,8%). Embora 70,9% das mulheres não tenham planejado a gestação, apenas uma referia não ter realizado o pré-natal. Na EPDS, respondida com um mês após o parto, 29,2% das puérperas apresentaram escore de risco para provável depressão puerperal. Em relação aos bebês, houve um discreto predomínio do sexo feminino (55,1%), com metade nascendo de parto normal (50,4%) e peso adequado (média de 3,3 kg), sendo apenas quatro (3,1%) prematuros (Tabela 1).

**Tabela 1:** Caracterização da amostra (N=127). São Paulo, SP, Brasil, 2021

CARACTERÍSTICAS MATERNAS		
Idade (anos)	**Mín.-Máx.**	**Média (DP) - Mediana (IC95%)**
	18 - 43	27,6 (6,0) - 28 (26,5 - 28,6)
Escolaridade	**N**	**%**
Até ensino fundamental	13	10,2
Ensino médio	90	70,9
Ensino superior	24	18,9
Estado civil	**N**	**%**
Sem companheiro	35	27,6
Com companheiro	92	72,4
Gestação planejada	**N**	**%**
Sim	37	29,1
Não	90	70,9
Fez pré-natal	**N**	**%**
Sim	126	99,2
Não	1	0,8
Paridade	**N**	**%**
Primíparas	51	40,2
Multíparas	76	59,8
Pontuação na EPDS	**Mín.-Máx.**	**Média (DP) - Mediana (IC95%)**
	0 - 26	7,8 (5,3) - 7,0 (6,9 - 8,8)
Classificação na EPDS	**N**	**%**
Normal	89	70,0
Provável depressão puerperal	37	29,2
Sem informação	1	0,8
**CARACTERÍSTICAS DO RECÉM-NASCIDO E CONDIÇÕES DE NASCIMENTO**		
Sexo	**N**	**%**
Masculino	57	44,9
Feminino	70	55,1
Tipo de parto	**N**	**%**
Normal	64	50,4
Cesárea	63	49,6
Idade gestacional	**N**	**%**
Termo	121	95,3
Pré-termo	4	3,1
Sem informação	2	1,6
Peso de nascimento (kg)	**Mín.-Máx.**	**Média (DP)**
	2,3 - 4,3	3,3 (4,0)
Uso de chupeta no primeiro mês de vida	**N**	**%**
Sim	51	40,2
Não	76	59,8

*Nota: DP - desvio padrão; IC95% - Intervalo de Confiança de 95%; Mín.-Máx. - Mínimo-Máximo; EPDS - Edinburgh Postnatal Depression Scale.*

A maioria das mães recebeu orientações sobre o aleitamento materno no alojamento conjunto (83,5%) e não durante o pré-natal (21,2%), e apenas 36,2% dos recém-nascidos foram amamentados na sala de parto. Quase metade dos recém-nascidos apresentou dificuldade para mamar no alojamento conjunto (48%), com melhora desse porcentual durante o primeiro mês de vida (17,3%), sendo que apenas sete necessitaram receber fórmula na maternidade. Apenas 63,8% dos recém-nascidos estavam em aleitamento materno exclusivo ao final do primeiro mês de vida, sendo que a fórmula foi introduzida antes dos 6 meses de idade em 48,8% dos bebês. O tempo total de aleitamento materno foi de 5,2 meses, sendo de 3,2 meses quando considerado aleitamento materno exclusivo (Tabela 2). A Figura 1 traz a evolução do tempo de aleitamento materno na amostra estudada.

**Tabela 2:** Práticas alimentares e tempo de aleitamento materno. São Paulo, SP, Brasil, 2021

Mãe recebeu orientações sobre aleitamento materno durante o pré-natal?	N	%
Sim	27	21,2
Não	100	78,8
Mãe recebeu orientações sobre aleitamento materno no alojamento conjunto?	**N**	**%**
Sim	106	83,5
Não	21	16,5
Recém-nascido mamou na sala de parto?	**N**	**%**
Sim	46	36,2
Não	81	63,8
Recém-nascido teve dificuldade para mamar no alojamento conjunto?	**N**	**%**
Sim	61	48,0
Não	66	52,0
Recém-nascido recebeu fórmula no alojamento conjunto?	**N**	**%**
Sim	7	5,5
Não	120	94,5
Recém-nascido apresentou dificuldade para mamar no primeiro mês de vida?	**N**	**%**
Sim	22	17,3
Não	105	82,7
Aleitamento materno no primeiro mês de vida	**N**	**%**
Exclusivo	81	63,8
Desmame parcial	45	35,4
Desmame total	1	0,8
Idade da introdução da fórmula	**N**	**%**
< 3 meses	17	13,4
≥ 3-6 meses	45	35,4
≥ 6 meses/não introduziu	65	51,2
Tempo total de aleitamento materno (meses)	**Mín.-Máx.**	**Média (DP) - Mediana (IC95%)**
	0->6	5,2 (1,5) - 6,0 (4,9 - 5,4)
	**N**	**%**
< 3 meses	11	8,7
≥ 3-6 meses	24	18,9
≥ 6 meses	92	72,4
Tempo de aleitamento materno exclusivo (meses)	**Mín.-Máx.**	**Média (DP) - Mediana (IC95%)**
	0 - 6	3,2 (2,3) - 4,0 (2,8 - 3,6)
	**N**	**%**
< 3 meses	50	39,4
≥ 3-6 meses	28	22,0
≥ 6 meses	49	38,6

*Nota: DP - desvio padrão; IC95% - Intervalo de Confiança de 95%; Mín.-Máx. - Mínimo-Máximo.*

**Figura 1:** Evolução do tempo de aleitamento materno (N=127). São Paulo, SP, Brasil, 2023

A partir da análise inferencial, observaram-se os fatores associados ao tempo de aleitamento, como o uso de chupeta no primeiro mês de vida (p=0,019), dificuldade para mamar no primeiro mês de vida (p=0,015), desmame precoce e introdução precoce de fórmula (p<0,001) (Tabela 3). A Figura 2 representa a correlação entre o tempo de aleitamento materno total e os escores de vínculo obtidos pelo PBQ no primeiro (p=0,518) e sexto mês de vida (p= 0,102).

**Tabela 3:** Fatores associados ao tempo de aleitamento total (N=127). São Paulo, SP, Brasil, 2021

	< 3 meses	≥ 3-6 meses	≥ 6 meses	Coeficiente	Valor de *p*
Idade materna	-	-	-	0,097	0,182
Escolaridade					
Até ensino fundamental	1(7,7)	3(23,1)	9(69,2)	0,058	0,491
Ensino médio	8(8,9)	18(20,0)	64(71,1)		
Ensino superior	2(8,3)	3(12,5)	19(79,2)		
Estado civil					
Sem companheiro	3(8,6)	9(25,7)	23(65,7)	-0,081	0,348
Com companheiro	8(8,7)	15(16,3)	69(75,0)		
Gestação planejada					
Sim	2(5,4)	4(10,8)	31(83,8)	0,155	0,073
Não	9(10,0)	20(22,2)	61(67,8)		
Fez pré-natal					
Sim	11(8,7)	23(18,3)	92(73,0)	-	-
Não	0	1(100,0)	0		
Paridade					
Primíparas	4(7,8)	8(15,7)	39(76,5)	0,069	0,427
Multíparas	7(9,2)	16(21,1)	53(69,7)		
Não	1(5,9)	4(23,5)	12(70,6)		
Classificação da EPDS					
Normal	9(10,1)	19(21,4)	61(68,5)	0,125	0,152
Provável depressão puerperal	2(5,4)	5(13,5)	30(81,1)		
Sexo					
Masculino	5(8,8)	11(19,3)	41(71,9)	-0,010	0,911
Feminino	6(8,6)	13(18,6)	51(72,8)		
Tipo de parto					
Normal	4(6,2)	16(25,0)	44(68,8)	-0,059	0,498
Cesárea	7(11,1)	8(12,7)	48(76,2)		
Idade gestacional					
Pré-termo	0	1(25,0)	3(75,0)	-	-
Termo	11(9,1)	22(18,2)	88(72,7)		
Uso de chupeta no primeiro mês de vida					
Sim	6(11,8)	14(27,4)	31(60,8)	-0,203	0,019
Não	5(6,6)	10(13,1)	61(80,3)		
Orientação sobre aleitamento materno no pré-natal					
Sim	2(7,4)	7(25,9)	18(66,7)	-0,055	0,525
Não	9(9,0)	17(17,0)	74(74,0)		
Orientação sobre aleitamento materno no alojamento conjunto					
Sim	8(7,6)	21(19,8)	77(72,6)	0,024	0,784
Não	3(14,3)	3(14,3)	15(71,4)		
Amamentação na sala de parto					
Sim	3(6,5)	11(23,9)	32(69,6)	-0,033	0,703
Não	8(9,9)	13(16,0)	60(74,1)		
Dificuldade para mamar no alojamento conjunto					
Sim	8(13,1)	12(19,7)	41(67,2)	-0,124	0,152
Não	3(4,5)	12(18,2)	51(77,3)		
Dificuldade para mamar no primeiro mês de vida					
Sim	6(27,3)	4(18,2)	12(54,5)	-0,211	0,015
Não	5(4,8)	20(19,0)	80(76,2)		
Aleitamento materno exclusivo no primeiro mês de vida					
Sim	2(2,5)	12(14,8)	67(82,7)	-0,317	<0,001
Não	9(19,6)	12(26,1)	25(54,3)		
Idade de introdução de fórmula					
< 3 meses	10(58,8)	4(23,6)	3(17,7)	-0,640	<0,001
≥ 3-6 meses	0	20(44,4)	25(55,6)		
≥ 6 meses/não introduziu	1(1,5)	0	64(98,5)		

*Nota: EPDS - Edinburgh Postnatal Depression Scale.*

**Figura 2:** Correlação entre o tempo de aleitamento materno total e os escores de vínculo mãe-bebê no primeiro (A) e sexto mês de vida (B) (N=127). São Paulo, SP, Brasil, 2021

## DISCUSSÃO

Neste estudo, 72,4% das mães mantinham o aleitamento materno até o sexto mês de vida da criança, porém apenas 38,6% estavam em aleitamento materno exclusivo, como o preconizado. Essa taxa está abaixo dos 45,8% descritos no Estudo Nacional de Alimentação e Nutrição Infantil (ENANI)^(25)^, do indicador global de 2020 (44%) e muito abaixo da meta preconizada pela Organização Mundial da Saúde (OMS), de 50%, em 2025, e 70%, em 2030^(26)^, mostrando falhas na promoção do aleitamento materno, considerado um dos pilares para o alcance dos ODS, particularmente dos ODS 2 e 3, relacionados ao estado nutricional e de saúde dos indivíduos, mas indiretamente também aos ODS relacionados a questões socioeconômicas e ambientais (ODS 5, 12 e 13).

Outro resultado relevante foi a elevada porcentagem de crianças (36,2%), que já no primeiro mês de vida, não estavam em aleitamento materno exclusivo, principalmente porque esse resultado refletiu no tempo total de aleitamento materno. Enquanto 82,7% das crianças em aleitamento materno exclusivo no primeiro mês de vida tiveram tempo de aleitamento materno total maior do que seis meses, apenas 54,3% das que não estavam receberam leite materno por mais de seis meses (p<0,001). É importante ressaltar que 46 crianças não estavam em aleitamento materno exclusivo no primeiro mês de vida, mas o consumo da fórmula antes dos três meses foi referido por um número bem menor (17 mães). Esse dado nos mostra que o desmame pode não ter sido devido ao uso precoce de fórmulas, mas sim pela introdução de outros líquidos, como água, suco e chá, e que isso acabou interferindo no tempo total de aleitamento materno. Essa é uma prática frequente e que deve ser desestimulada. Resultado semelhante foi descrito por outros autores, e uma das explicações seria a interferência no hábito da sucção^(27)^. Possivelmente, problemas com a técnica de amamentação foram um dos principais motivos para o desmame precoce, pois o relato de dificuldade de mamar no primeiro mês de vida também esteve associado ao menor tempo total de aleitamento materno (p=0,015). O aleitamento materno exclusivo até os seis meses de idade tem sido associado à redução da morbimortalidade materno-infantil^(5-8)^. Portanto, o desmame precoce identificado neste estudo pode representar uma ameaça ao alcance dos ODS 2 e 3.

Além de se associar ao desmame precoce, a oferta de águas, sucos e chás não atende às necessidades nutricionais do recém-nascido e lactente jovem, mas a aumenta o risco de contaminação, particularmente em condições de menor higiene^(28)^, comprometendo a segurança alimentar e estado nutricional (ODS 2)^(3)^. Esses resultados demonstram a importância da consulta de puericultura precoce nos primeiros dias após a alta da maternidade, para que seja avaliada a amamentação e que as mães sejam adequadamente orientadas quanto à técnica e à práxis de introdução de outros líquidos além do leite materno.

Orientar adequadamente e apoiar a mulher é essencial para o sucesso do aleitamento materno. O empoderamento feminino é correlacionado ao aleitamento materno como uma “via de mão-dupla”: mulheres que se sentem seguras têm maiores chances de sucesso na amamentação; por outro lado, o sucesso da amamentação estimula a autonomia materna^(9,10)^, promovendo o alcance do ODS 5. Nesse sentido, outro resultado que chama a atenção neste estudo é que, apesar de praticamente todas as gestantes terem realizado o pré-natal, a maioria (78,8%) referiu que não recebeu orientações sobre o aleitamento materno durante esse acompanhamento. Sabe-se que a decisão de amamentar a criança ou não ocorre, na maioria das vezes, bem antes do parto, e as orientações prestadas durante a assistência pré-natal contribuem para a decisão da mulher pelo aleitamento e para a sua duração^(29)^. Por outro lado, 83,5% das mães estudadas referiram terem sido orientadas pelo enfermeiro do alojamento conjunto, e isso pode ter influenciado a diminuição da porcentagem de recém-nascidos com dificuldade para mamar: de 48% na maternidade para 17,3% após a alta hospitalar. Essas orientações podem ter contribuído para o tempo total de aleitamento materno, embora não tenha sido encontrada significância estatística.

As reflexões supracitadas demonstram a necessidade de empoderamento dos serviços de saúde, especialmente a atenção primária, que possui papel ativo no pré-natal, na visita domiciliar e nas consultas de puericultura, e do empoderamento dos profissionais de saúde, fazendo uso desses dispositivos para a sua atuação na amamentação, impactando, consequentemente, os ODS.

Outro achado que reforça a importância das orientações e da relação entre práticas que interferem no hábito de sucção e aleitamento foi a associação de desmame precoce e o uso de chupetas no primeiro mês de vida (p=0,019). Neste estudo, 40,2% faziam uso de chupeta, semelhante aos 43,9% descritos no ENANI^(25)^. As chupetas são bicos artificiais feitos de borracha ou silicone que visam proporcionar conforto e satisfazer o reflexo de sucção em bebês. Seu uso tem sido associado a uma série de benefícios potenciais, incluindo a redução do risco de síndrome da morte súbita infantil e alívio da dor durante manipulações invasivas. No entanto, a chupeta tem sido associada a resultados negativos, incluindo a diminuição das taxas e duração da amamentação^(30)^, como visto nos resultados. Pesquisas sugerem que o uso de chupeta pode interferir na capacidade do bebê de sugar com eficácia, levando à confusão de bicos, diminuição da produção de leite e menor duração da amamentação^(31)^, porém esses achados ainda são inconsistentes. Outros autores não constataram esse impacto no uso de chupeta nos resultados da amamentação^(32)^. De qualquer forma, a Academia Americana de Pediatria recomenda adiar o uso da chupeta até que a amamentação esteja bem estabelecida, geralmente por volta de 3-4 semanas de idade, assim como a Iniciativa Hospital Amigo da Criança, programa global para incentivo à amamentação que também desestimula o uso rotineiro de chupeta em lactentes^(33)^.

Quanto à introdução precoce de fórmula, também encontramos associação ao tempo total de aleitamento (p<0,001). Aqui, vale considerar que é difícil identificar o que foi causa e o que foi consequência, ou seja, se as crianças que receberam fórmula mamaram por menos tempo ou vice-versa. De qualquer forma, esse é um achado também discutido na literatura^(34)^, particularmente quando essa introdução é muito precoce, nos primeiros dias de vida.

Investigação prévia constatou que a introdução de fórmula no hospital foi associada a uma menor duração do aleitamento materno exclusivo. As justificativas relatadas incluem interferência na capacidade de sucção, redução na produção láctea devido à menor frequência das mamadas e possível alteração na percepção materna sobre a suficiência do leite materno, uma vez que elas tendem a considerar seu suprimento de leite como inadequado e interromper a amamentação precocemente^(34)^. Aqui, não foi possível analisar o impacto da introdução nos três primeiros dias de vida, como descrito por alguns autores, porque houve um número reduzido de crianças que receberam fórmula na maternidade (5,5%). Contudo, verificou-se que quanto mais precoce a introdução da fórmula, menor o tempo total de aleitamento materno.

Apesar de não ter sido observada associação entre o desmame precoce e a presença de provável depressão puerperal, como frequentemente relatado na literatura, chama a atenção a porcentagem elevada (29,2%) de puérperas com escore de risco. Investigação realizado antes da pandemia, envolvendo 2.687 mulheres e utilizando o mesmo instrumento de rastreamento e pontuação de corte, descreveu prevalência de 14%^(35)^. Aqui, os resultados indicaram que o risco de depressão puerperal dobrou durante a pandemia. Outros autores^(36)^ encontraram taxas ainda mais elevadas no Brasil (38,8%) e na Espanha (37,3%)^(37)^. Esses achados alertam para a necessidade de um olhar mais cauteloso e atento para essa questão por parte dos profissionais de saúde que atendem puérperas e lactentes. A incorporação rotineira de escalas de triagem para a depressão materna nos protocolos de atendimento deve ser considerada pelos serviços de saúde. Essa piora da saúde mental foi generalizada durante a pandemia, sendo um dos pontos considerados mais impactantes para o alcance do ODS 3^(12,13)^.

A depressão puerperal é associada a vários desfechos negativos, como a piora do vínculo mãe-bebê. Em nosso estudo, 9,4% da amostra apresentou pontuação alterada no PBQ realizado no primeiro mês de vida, indicando que o vínculo entre a díade esteve alterado precocemente. Possivelmente, esse achado se deve aos efeitos negativos da pandemia sobre a saúde mental. Estudo que comparou duas amostras de mães antes e durante a pandemia encontrou 2,56 vezes mais chances de escore alterado na avaliação do vínculo um mês após o parto^(38)^. Esse estudo corrobora outra investigação, que encontrou piora nos escores do PBQ realizado no primeiro ano de vida em mães portuguesas, relacionando os piores escores ao estresse parental^(39)^. Aqui, os pesquisadores não dispunham de dados anteriores à pandemia, não sendo possível afirmar que houve uma relação de causa-efeito. Entretanto, a elevada prevalência de depressão puerperal pode explicar esse resultado desfavorável. Outro aspecto que poderia explicar esses escores alterados de PBQ no primeiro mês de vida é o fato de que apenas 36,2% dos recém-nascidos foram amamentados na sala de parto. Sabe-se que esse contato pele a pele é amplamente difundido e estimulado para favorecer o vínculo entre mãe-filho^(40)^, mas devemos considerar que este estudo ocorreu na fase de maior restrição, durante a pandemia, em que os protocolos assistenciais em sala de parto tiveram que ser modificados.

Embora não tenha apresentado significância estatística, observou-se uma correlação entre o escore do PBQ, tanto no primeiro quanto no sexto mês de vida, e o tempo de aleitamento materno, indicando que o maior vínculo esteve associado ao maior tempo de amamentação. A falta de significância estatística aponta que a relação entre ambos é complexa e que múltiplos fatores estão envolvidos no estabelecimento do vínculo mãe-filho e para o sucesso do aleitamento materno.

Em 2018, a OMS divulgou a iniciativa *Nurturing Care*, que deve guiar os cuidados à saúde da criança. Entre os seus componentes essenciais, há a promoção da saúde, nutrição adequada, cuidado responsivo, segurança e fornecimento de oportunidades para o desenvolvimento infantil. Esses componentes estão interligados com os 17 ODS, sendo central para a reflexão de estratégias para o desenvolvimento sustentável^(41)^. O aleitamento materno se integra a todos os componentes supracitados, como indicado ao longo deste manuscrito. Ameaças à sua manutenção podem impactar a saúde global de crianças e, consequentemente, a humanidade, reforçando a importância do olhar ao fenômeno e guiando novas pesquisas que visem modificar esse cenário.

Reitera-se que, neste estudo, embora a maior parte das crianças tenha recebido aleitamento materno por seis meses ou mais, a taxa de desmame precoce, desde o primeiro mês, foi muito alta. Esse resultado pode representar barreiras para se atingir as metas dos ODS, especialmente a melhor nutrição e menor insegurança alimentar (ODS 2), melhor resultado em saúde e bem-estar (ODS 3), promoção à equidade de gênero (ODS 5), consumo responsável (ODS 12), e mudança climática global (ODS 13).

### Limitações do estudo

Por se tratar de estudo de acompanhamento a distância durante seis meses realizado na pandemia, a perda amostral pode caracterizar um viés de seleção e uma limitação, uma vez que, possivelmente, as perdas foram de mães com piores resultados, por não responderem aos questionários. Outra limitação é a ausência de possibilidade de estabelecimento de relações de causalidade dos achados com a pandemia e relações diretas com os ODS. Apesar disso, foi possível caracterizar a prática de aleitamento materno, a provável depressão puerperal e o vínculo no período mais crítico desse momento histórico, com discussões diante dos ODS.

### Contribuições para as áreas da enfermagem, saúde ou políticas públicas

Este estudo demonstra a importância do cuidado materno-infantil e da necessidade de um olhar cauteloso e atento do enfermeiro e demais profissionais de saúde que atuam com gestantes, puérperas e lactentes. Os fatores associados ao desmame precoce possivelmente são reflexos da falta de orientação e apoio que as puérperas receberam durante a pandemia, devido ao distanciamento social e menor acesso às consultas de puericultura. Esses achados demonstram que, para promover o aleitamento materno exclusivo, um importante pilar para o alcance dos ODS, são necessárias medidas factíveis, por meio de uma assistência materno-infantil de qualidade. Ainda, destaca-se o caráter de inovação deste manuscrito, por atuar sobre um fenômeno de importância internacional em um período crítico à saúde pública e por suas articulações com os ODS, demonstrando que, mesmo com o fim do período pandêmico, esforços devem ser conduzidos para mitigar os eventuais impactos a longo prazo proporcionados por esse contexto.

## CONCLUSÕES

No primeiro semestre de vida, o tempo total de aleitamento materno foi de 5,2 meses, e de aleitamento materno exclusivo, 3,2 meses. Embora 72,4% mantivessem o aleitamento materno até o sexto mês de vida, apenas 36,2% estavam em aleitamento exclusivo no primeiro mês de vida, caracterizando um elevado porcentual de desmame precoce durante a pandemia de COVID-19. Esses resultados apontam para maiores riscos nutricionais e de saúde entre essas crianças nascidas durante a pandemia e, portanto, com possível impacto para o alcance dos ODS 2 e 3. Da mesma forma, o desmame precoce verificado pode, indiretamente, prejudicar o alcance dos ODS 5, 12 e 13, ao reduzir os benefícios socioeconômicos e ambientais associados à prática da amamentação.

Ao avaliar os fatores que estiveram associados ao menor tempo de aleitamento materno total, este estudo traz reflexões para melhorar a prática assistencial. Os fatores identificados foram uso de chupeta (p=0,019), dificuldade para mamar (p=0,015), ambos no primeiro mês de vida, desmame precoce (p=0,001) e introdução precoce de fórmula (p=0,001), reforçando a importância de aperfeiçoamento de uma assistência voltada à promoção do aleitamento materno, no sentido de mitigar os efeitos indesejados para o alcance dos ODS no período pós-pandêmico.

## Data Availability

Os dados de pesquisa só estão disponíveis mediante solicitação.
